# Predictors of time to full enteral feeding in low birth weight neonates admitted to neonatal intensive care unit: a prospective follow up study

**DOI:** 10.1186/s12887-024-04545-0

**Published:** 2024-01-20

**Authors:** Abraraw Terefe, Asrat Demtse, Fikertemariam Abebe, Esuyawkal Mislu, Erdaw Tachbele

**Affiliations:** 1 Midwifery Department, College of Health Science, Woldia University, Weldiya, Ethiopia; 2 College of Medical Science, Addis Ababa University, Addis Ababa, Ethiopia; 3 College of Health Science, School of Nursing, Addis Ababa University, Addis Ababa, Ethiopia; 4 Nursing & Midwifery, College of Health Sciences, Addis Ababa University, Addis Ababa, Ethiopia

**Keywords:** Ethiopia, Full enteral feeding, Low birth weight, Predictors, Preterm

## Abstract

**Background:**

Survival of LBW infants has increased in recent years because of novel perinatal interventions, but the introduction and advancement of enteral feeds for low birth weight infants is challenging. In Ethiopia the proportion of low birth weight infants is thought to be 17.3%. The purpose of this study was to determine the time to full enteral feeding (FEF) and its predictors in LBW neonates admitted to neonatal intensive care unit in selected hospitals of Addis Ababa, Ethiopia.

**Method:**

An institutional based prospective follow up study was conducted from March 15 to June 15, 2022 among 282 LBW neonates admitted to six randomly selected hospitals. Both primary and secondary data was used by interviewing mothers and prospective medical chart review of neonates. The Cox regression model was used and variables having a *p*-value less than 0.05 with 95% CIs in a multivariable analysis were declared as statistically significant association with time to full enteral feeding.

**Result:**

Out of 282 neonates involved in this study, 211 (74.8%) of them reached at FEF. The overall median time to full enteral feeding was 5 days. Predictors significantly associated with time to full enteral feeding were educational level, birth weight, cesarean delivery, hospital acquired infection, being on antibiotics, age at initiation of trophic feeding, routine gastric residual evaluation and NICU location (hospital).

**Conclusions:**

This study demonstrated the difficulty of understanding which low birth weight neonate will attain FEF in a timely manner and factors that affect time to FEF. There is a delay in full enteral feeding achievement among low birth weight neonates and there is a great deal of heterogeneity of practice among health care providers regarding feeding of infants as it was evidenced by a variation in feeding practice among hospitals. Nutrition should be considered as part of the management in neonatal intensive care units since low birth weight neonates are developing edematous malnutrition while they are in the NICU. There should be standard feeding protocol to avoid heterogeneity of practice and additional study should be conducted for each categories of GA and BW with long follow up time.

## Background

Infant and young child feeding (IYCF) is fundamental for infant and child survival, healthy growth and development, a healthy future generation and national development [1]. The United Nations (UN) Convention on Child Rights declared that every infant and child has the right to good nutrition [2].

Since LBW (low birth weight) infants had an interrupted growth trajectory, immediate nutrition after birth is important to avoid postnatal delay in nutritional intake and growth restriction over time [3]. According to a prospective study done in Ethiopia in 2020 majority of the neonates (86.2%) had extra uterine growth restriction (EUGR) at the time of discharge from the hospital, which indicates suboptimal nutrition during their stay in the neonatal intensive care unit [4]. The problem affects the most vulnerable groups of newborns like LBW (low birth weight) and small for gestational age neonates which aggravates complications associated with their prematurity.

In addition to its nutritional benefits human milk have an increasingly central role in immune protection and intestinal maturation of a highly vulnerable population such as severely preterm infants [3]. LBW and critically ill infants have an increased metabolic needs and minimal macronutrient stores and this increases their energy requirement and evidence supports that early enteral feeding affect important outcomes like enhanced neurodevelopmental outcome and growth and development [5, 6]. But in spite of these benefits, late breast feeding initiation, low breastfeeding rates and short breastfeeding duration is common in LBW infants [5].

Enteral nutrition is preferred over total parenteral nutrition (TPN), because it avoids complications related to vascular catheterization, sepsis and adverse effects of TPN [7]. Early full enteral feeding also increase nutrient intake and growth rates, accelerate time to achieve full enteral feeding, and prevent late-onset sepsis (LOS) in infants [8]. Late attainment of full enteral feeding in LBW neonates will prolong hospitalization and healthcare costs and suboptimal nutrition in these groups of neonates will lead to decreased survival, poor childhood growth and it will have a negative effect on healthy future generation and national development [1].

In recent years, much has been researched on possible short and long-term health consequences related to under nutrition and important results have been achieved with regard to nutrition in neonates [6]. Parenteral nutrition, enriched preterm formula, and fortification of human milk have been proven to be critically important for LBW infants admitted to neonatal intensive care units [9]. But these nutrition options are usually not available in low-resource countries, and unfortified human breast milk is the only option available in many low-income countries and varying degrees of protein energy malnutrition are very common [10].

The only parenteral source of nutrition for infants admitted to our unit, like many units in sub-Saharan Africa, is 10% dextrose solution with or without sodium chloride. This solution is usually reconstituted in the ward with potassium chloride and calcium to provide the infant’s daily recommended requirements until they can tolerate enteral feeds. The use of total parenteral nutrition can be limited by feasibility and affordability and human breast milk is practically feasibly strategy of feeding LBW infants in countries with low socioeconomic status like Ethiopia.

Currently, in most settings in resource-limited countries, there is inevitable suboptimal feeding of LBW infants. Unlike the general recommendation to initiate early enteral feeding, a considerable number of the infants were kept NPO (nothing by mouth) in the first few days, receiving only maintenance fluid. This was associated with increased risk of death and development of hypoglycemia [11]. Therefore, the purpose of this study was to estimate time to full enteral attainment and its predictors among low birth weight neonates admitted to selected public hospitals of Addis Ababa.

## Methods

### Study design and setting

Institution based multicenter prospective follow up study was conducted in NICU (Neonatal intensive care unit) of six randomly selected public hospitals in Addis Ababa, Ethiopia, from March to June 2022. The city has twelve public hospitals among these, six hospitals (Gandhi Memorial Hospital, Yekatit 12 Hospital Medical College, Zewditu Memorial Hospital, Ras Desta Damtew Memorial Hospital, Menelik II Referral Hospital and Tirunesh Beijing General hospital) are governed by Addis Ababa City Health Bureau and the rest five (St Peter Specialized Hospital, St’ Paul’s Hospital Millennium Medical College, Amanuel Hospital, Alert Hospital and Eka Kotebe Hospital) are governed by federal ministry of health and one university hospital (Tikur Anbessa Specialized Hospital). All hospitals except Amanuel Hospital, have their own neonatal intensive care unit. So, the study was conducted on NICUs located in Gandhi Memorial Hospital, Zewditu Memorial Hospital, Menelik II Referral Hospital, St Peter Specialized Hospital, St’ Paul’s Hospital Millennium Medical College and Tikur Anbessa Specialized Hospital. Each selected low birth weight infants with their indexed mothers were the study units.

### Study population

The source population comprises all low birth weight neonates and the study population includes all randomly selected low birth weight neonates and their respective care takers during the study period.

### Inclusion and exclusion criteria

All low birth weight neonates admitted to NICU in selected hospital with in the study period were included and neonates with major congenital cardiac disease, gastro intestinal defect, necrotizing enterocolities, and neonates who died in their immediate postnatal day were excluded.

### Sample size and procedure of sampling

To determine the required sample size, different factors which were significantly associated with the time to full enteral feeding in low birth weight neonates were considered with the following assumption; 95% confidence level, 80% power, margin of error = 5%=0.05 and maximum sample size (278) was taken for the required sample size. Double population proportion formula was used by considering study in Auckland, New Zealand [12], by taking gestational age as a predictor variable. The sample size was calculated using STATA version 14.

**Fig. 1:**
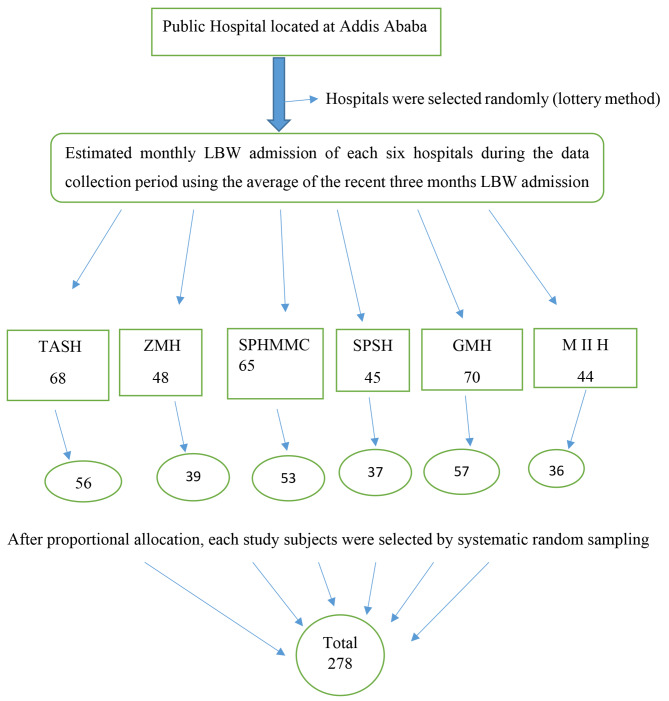
Schematic representation of sampling procedures of the study, Addis Ababa, Ethiopia, March 2022 G.C.

Six hospitals were selected by lottery method. To estimate the total source population during the data collection period, monthly average low birth weight admission was estimated based on the data taken from the recent three months HMIS registration of each selected hospital. The estimated source population was 340 and the k value was 1.22. Therefore, by using systematic sampling technique, four from each five consecutive low birth weight admissions that fulfill the inclusion criteria were selected randomly throughout the study period in each hospital (Fig. 1).

### Operational definitions

Neonate: A neonate is a child under 28 days of age.

Low birth weight: Babies who are born weighing less than 2,500 g [13].

Very low birth weight: Babies who are born weighing less than 1,500 g [14].

Full enteral feeding: Newborn infants receive all of their nutrition as milk feeds (either human milk or formula) orally [15] and total enteral intake of 150 ml/kg/day or at least 110 Kcal per kg per day sustained for 24 h sustained for 24 h through tube feeding [16].

Time to event: Time from birth of a low birth weight infant to full attainment of enteral feeding.

Censored: LBW neonates who left the study before reaching at the event of interest (FEF) or does not attain full feeding before the study ends (28 days).

Event: Refers to the occurrence of the outcome of interest (attainment of FEF).

Follow up time: Time from recruiting up to either the study subjects attains full enteral feeding or censored.

Trophic feeding: The volume of feeding considered trophic in most neonatal nurseries is 12 cc/Kg/d or less [17].

Survival time: the length of time in days followed starting from birth to full enteral feeding.

### Data Collection tools and procedure

Interviewers administered structured questionnaire was used to collect the data. The data extraction tool was adapted from previous related studies [18–29] and modified to local context.

#### Data quality control

Six data collectors were recruited based on their experience in data collection, qualification and ability of the local language. A one day training was given to data collectors and the supervisor regarding significance of the study and ways of data collection process. Data extraction form was checked before data collection for completeness and consistency using 14 LBW neonates from Ras Desta Hospital and faults found during the process were corrected by the principal investigator. Data was examined for completeness and consistency during data cleaning, storage, and analysis.

### Data processing and analysis

Time to full enteral feeding attainment was calculated in days using the time interval between the time of birth and the time of full enteral feeding attainment. Data was entered and cleaned by using EPi Data version 4.6 and transported to STATA version 14 for analysis. The Kaplan Meier failure method was used to estimate the cumulative probability of full enteral feeding, and a log-rank test was used to compare the time to event curves of categories of variables. Bi-variable Cox-regression was computed for each predictor variable, and variables with *p*-value of < 0.25 were entered to multivariable Cox-regression and the significant association was declared with a *p*-value less than 0.05 in a multivariable Cox regression model.

## Results

### Socio-demographic characteristics the study participants

**Fig. 2:**
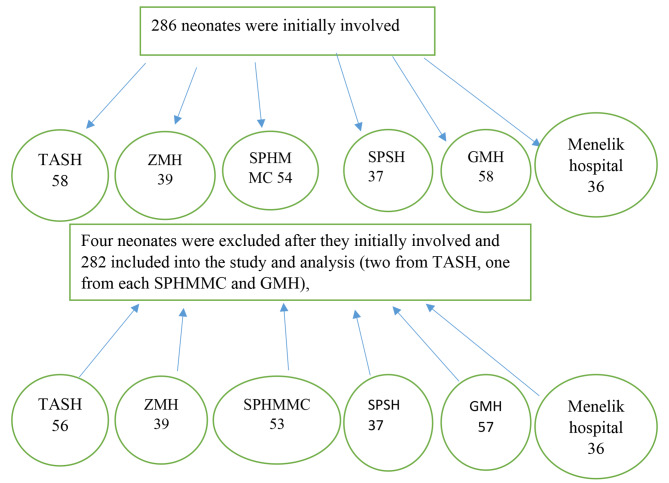
Diagrammatic presentation of the number of neonates initially to the study and numbers of neonates finally included into the study and analysis from each hospitals

A total of 286 neonates were involved initially but four neonates were diagnosed with the exclusion criteria after they had been recruited to the study. Response was obtained from 282 (response rate of 98.6%) participants (Fig. 2). The mean age of indexed mothers was 27.27 ± Std. Dev. of 4.88 years and 242 (85.82%) were married. The maximum and minimum maternal ages found in this study were 17 and 40 respectively. 82(29%) mothers had no formal education and 50(17.73%) have higher educational level. Most 216 (76.6%) mothers were urban residents.

### Maternal prenatal, medical and obstetrics related characteristics

88 (31.2%) of mothers were found to have at least one pregnancy related complications and the major reported complication was premature rupture of membrane (21.6%). Majority, 276 (97.8%) of the mother gives birth at health institution. 146 (51.7%) of mothers were multipara.

### Neonatal characteristics

The minimum and maximum GA at birth was 26 weeks and 41 weeks respectively. The mean GA was 33.58 ± 2.7 SD week and a 95% CI of 33.2–34 weeks respectively. The smallest and largest BW were 900 gram and 2490 gram respectively with a mean birth weight of 1729.7 ± SD 427.7 gram (95% CI of 1679.5–1780 gram) Table 1.

**Table 1:** Distribution of the neonatal characteristics of LBW neonates admitted in selected governmental hospital of Addis Ababa, Ethiopia 2022. (*N* = 282)

Variables	Categories	Total	Status	
		Number (%)	Reached FEF (%)	Censored (%)
Sex of neonate	Male	151 (53.5%)	114 (54%)	37 (52%)
	Female	131 (46.45%)	97 (46%)	34 (48%)
Gestational age	≤ 28 weeks	6 (2%)	1 (0.47%)	5 (7%)
	29–31 weeks	65 (23%)	40 (19%)	26 (36.6%)
	32–36 weeks	168 (60%)	134 (63.5%)	32 (45%)
	≥ 37 weeks	43 (15.2%)	35 (16.5%)	8 (11%)
Birth weight	1500–2499 gm.	192 (68%)	75 (35.5%)	17 (24%)
	1000–1499 gm.	81 (28.7%)	79 (37.4%)	21 (29.5%)
	< 1000 gm.	9 (3.2%)	0	9 (12.6%)
Birth Plurality	Single	220 (78%)	170 (80.6%)	50 (70.4%)
	Multiple	62 (22%)	39 (19.4%)	21(29.6%)
Weight for gestational age	SGA	74 (20.57%)	62 (29.3%)	12 (17%)
	AGA	206 (73.05%)	147 (69.6%)	59 (83%)
	LGA	2 (0.71%)	2 (1%)	0

AGA, Appropriate for gestational age; FEF, Full enteral feeding; LGA, large for gestational age; SGA, Small for gestational age

### Treatment and health service related predictors

Majority (87.6%) of neonates were on antibiotics and 224 (79.43%) needs respiratory support. 31.21% of indexed mothers reported that they did not receive instructions and counseling about feeding of their child (Table 2).

**Table 2:** Treatment related predictors among LBW neonates admitted to neonatal intensive care unit of Addis Ababa public hospitals, Ethiopia, 2022

Variables	Categories	Total	Status	
		Number (%)	Reached FEF (%)	Censored (%)
Respiratory support type	CPAP	140 (62.22%)	88 (41.7%)	52 (73%)
	INO2	85(37.78%)	69 (58.3%)	16 (27%)
Kangaroo mother care		91 (32.27%)	88 (41.7%)	3 (4.2%)
Trophic feeding initiated		204 (72.3%)	180 (85.3%)	24 (33.8%)
Milk type while on trophic feeding	Human milk	177 (86.7%)	154 (85.6%)	23 (95.8%)
	Formula	25 (12.25%)	24 (13.3%)	1 (4.2%)
	Mixed	2 (1%)	2 (1.1%)	0
TF interval	≤ 3 h	167 (81.8%)	150 (83.3%)	17 (70.8%)
	3–6 h0ur	32 (15.6%)	26 (14.4%)	6 (25%)
	Above 6 h	5 (2.5%)	4 (2.2%)	1 (4.2%)
Routine Evaluation of Gastric Residual		71 (34.8%)	59 (32.7%)	12 (50%)
Was Using Pacifier		13 (4.6%)	12 (5.6%)	1 (1.4%)

CPAP, continuous positive airway pressure; FEF, Full enteral feeding; INO2, Intranasal oxygen

### Time to attain full enteral feeding in low birth weight neonates admitted to neonatal intensive care unit

Two hundred eighty two LBW neonates have been followed to a total of 2179 neonate-days with a mean follow up days of 7.72 ± 5.97 days SD and a minimum of 1 day to a maximum of 28 days. Finally 211 (74.82%) neonates were attained FEF and the rest were censored with death being the primary cause 64 (90.14%). 46 (72%) of deceased neonates died without any enteral feeding attempts. The earliest time to reach at FEF was at first postnatal day while the latest time was at 28 days of post natal age. The median and mean age at FEF was 5 and 7.18 + 5.3 day (95% CI of 6.4–8 days) respectively. About 64.93% of neonates reached at FEF within 7 days of post natal age and 13.27% of LBW neonates reached FEF after 14 days.

### Kaplan-Meir survival estimates for the time to attain FEF

**Fig. 3:**
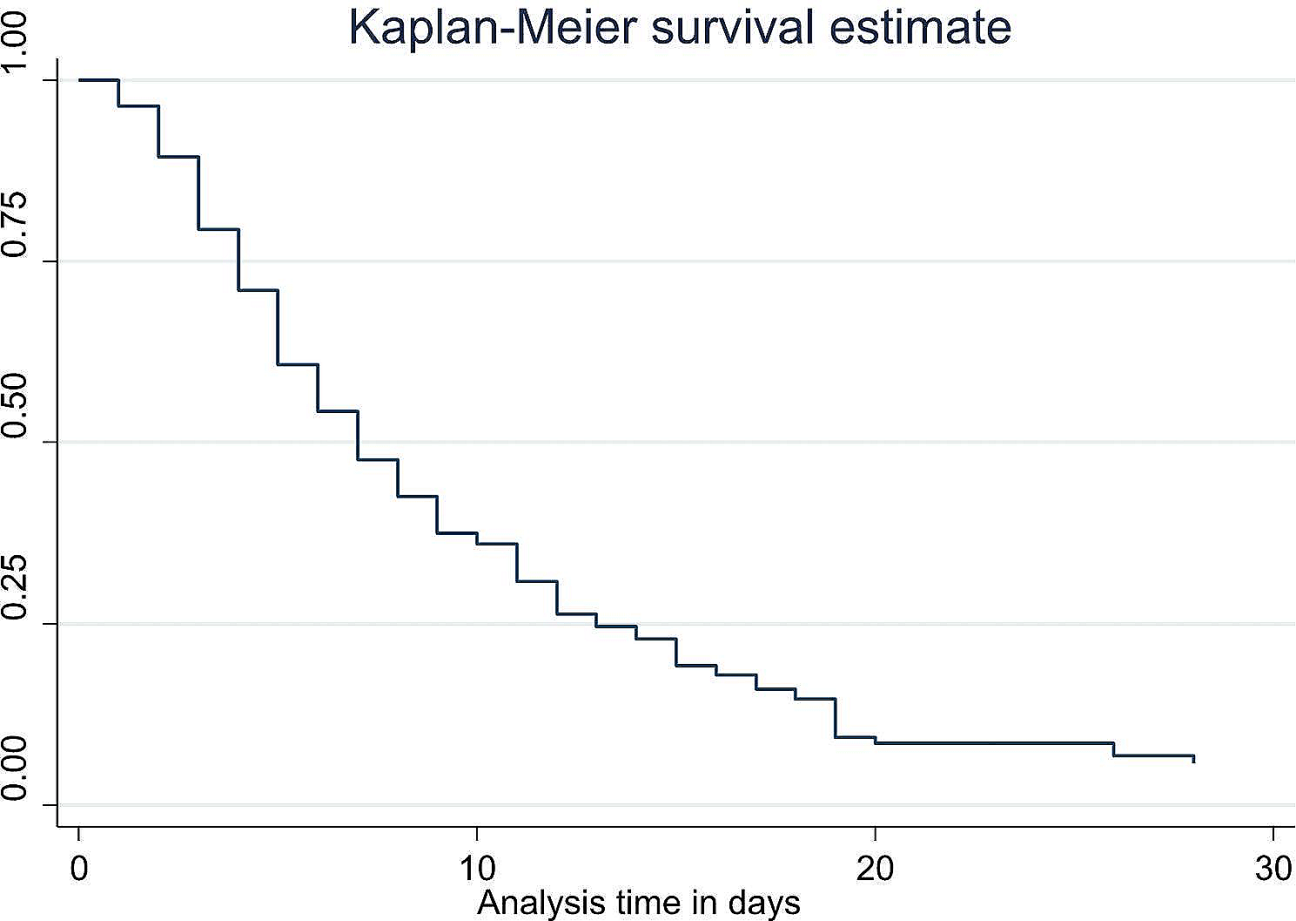
The Kaplan-Meier estimate of time to event (full enteral feeding) curve used to estimate the time to full enteral feeding of low birth weight neonates admitted to neonatal intensive care unit of Addis Ababa public hospitals, Ethiopia, 2022

Within the first week of postnatal age, the curve has a tendency to decline rapidly, implying that most low birth weight neonates attain full enteral feeding with in this time frame (Fig. 3).

### Comparison of time to FEF for different categorical variables

The median time to attain full enteral feeding (FEF) have a strong variation depending on the GA and BW of the neonates. Time to FEF is inversely related to both gestational age (GA) and birth weight (BW). Neonates whose GA is between 28 and 31 weeks have a median time to FEF of 11.5 days (95% CI: 9–14) and those neonates who born at a GA of between 32 and 37 weeks attains FEF at a median age of 5 days (95% CI: 5–6) while term born LBW neonates attains FEF at a median age of 3 postnatal days (95% CI: 2–3). Neonates whose BW was between 1500 and 2499 gram attains FEF at a median postnatal day of 4 while those whose BW was between 1000 and 1499 gram reached at FEF at their median age of 11 postnatal day (95% CI: 9–12) (Table 3).

**Table 3:** Median time to FEF and log-rank test for equality of survivor functions among LBW neonates admitted to NICU of Addis Ababa public hospitals, Ethiopia, 2022 (*n* = 282)

Variables	Category	Median time to FEF Point estimate(95%CI)	Log-rank x2 value	*p*-value
Parity	Primipara	6 (5–7)	2.05	0.15
	Multipara	5 (4–6)		
Gestational age in weeks	< 28	15 ( only 1 observation)	85	0.00
	28–31	12 (9–14)		
	32–36	5 (5–6)		
	Term	3 (2–3)		
Birth weight in gram	1500–2499	4 (4–5)	37	0.0000
	1000–1499	11 (9–12)		
	< 1000	No any		
Age at admission in hour	Within 1 h	7 (5–8)	12.01	0.007
	1–6 h	4.5 (4–5)		
	6–24 h	7 (5–19)		
	After 24 h	4 (3–6)		
Onset of labor	Spontaneous	5 (4–6)	3.82	0.05
	Induced	7 (5–8)		
Weight for gestational age	SGA	4 (3–6)	4.1	0.04
	AGA	6 (5–7)		
Apgar score	≤ 3	12	7.2	0.06
	4–6	7 (5–10)		
	≥ 7	5 (4–6)		
	unknown	6.5 (4–9)		
Plurality	Single	5 (5–6)	1.7	0.19
	Multiple	6 (4–11)		
Hypothermia	Yes	6 (5–6)	6.4	0.01
Respiratory distress	Yes	6 (5–7)	21	0.0000
New diagnosis during follow up	Yes	7 (6–9)	62	0.0000
Hospital acquired infection	Yes	11 (7–16)	22.4	0.0000
Necrotizing enterocolitis	Yes	16 (3 - )	10	0.001
Apnea during follow up	Yes	12 (10–19)	7.4	0.006
Thrombocytopenia in the follow up	Yes	9 (7–11)	17	0.0000
On antibiotics	Yes	6 (5–7)	49.8	0.0000
Respiratory support	Yes	7 (6–8)	64	0.0000
Type of respiratory Support	CPAP	9 (7–11)	17.4	0.000
	Intra nasal O2	5 (4–6)		
Feeding instruction provided	Yes	5 (5–6)	2.3	0.13
Trophic feeding	Yes	6 (5–7)	25	0.000
Age at trophic feeding initiation in hour	Within 24 h	3 (3–4)	31.92	0.0000
	24-48 h	5 (5–7)		
	48-72 h	8 (6–11)		
	After 72 h	13 (10–18)		
Trophic feeding interval	Every 2-3 h	5 (5–7)	1.2	0.55
	Every 3-6 h	7 (5–10)		
	Above 6 h	5 (5- )		
Routine gastric residual evaluation	Yes	9 (7–12)	23.46	0.0000
Milk type at trophic feeding	Human milk	6 (5–7)	8.4	0.015
	Formula	4 (3–7)		
	Mixed	5 (5- )		

AGA, Appropriate for gestational age; CPAP, continuous positive airway pressure; FEF, Full enteral feeding; SGA, Small for gestational age

**Fig. 4:**
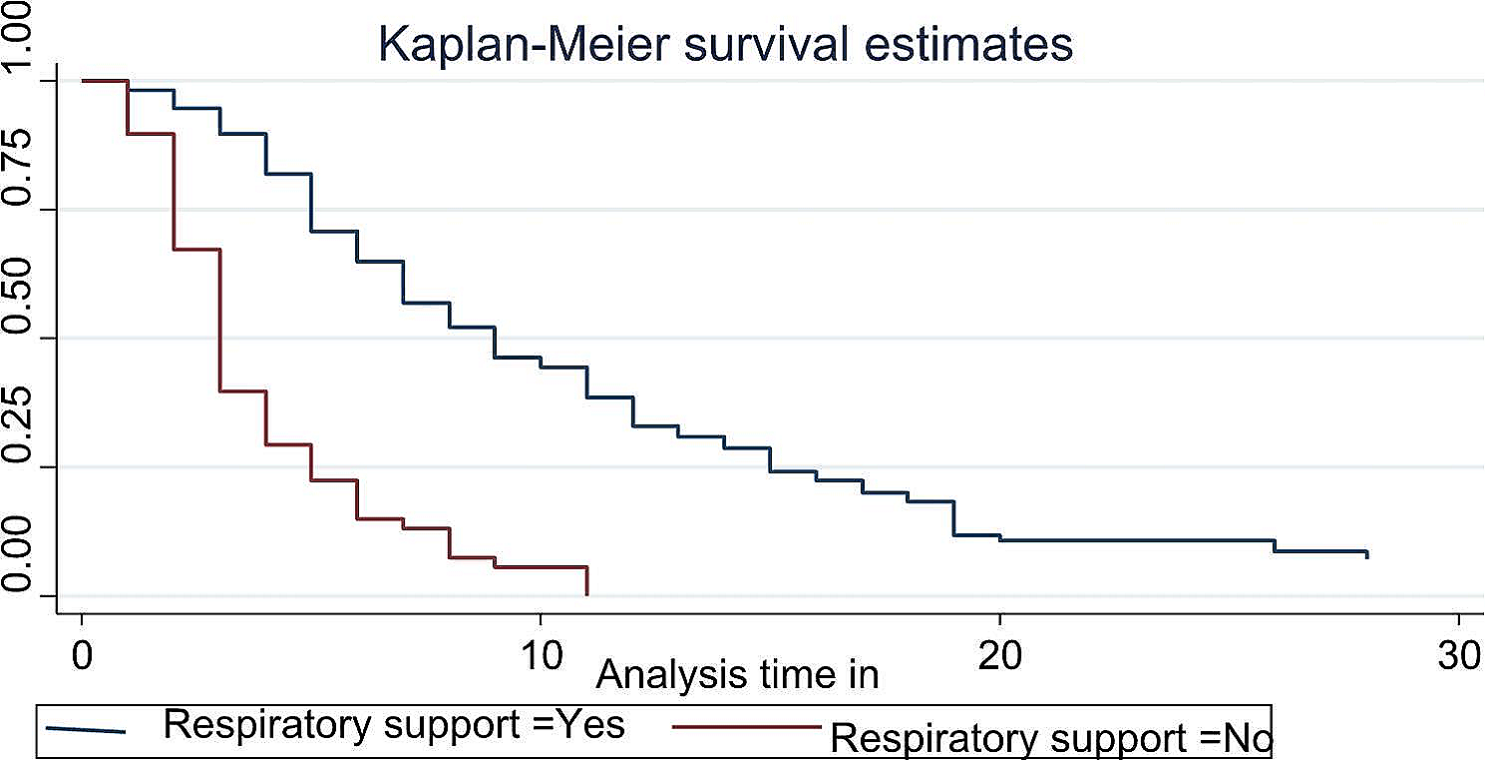
The Kaplan-Meier estimate of time to event (full enteral feeding) curve used to estimate the time to full enteral feeding of low birth weight neonates based respiratory support

No neonate with a BW of less than 1000 gram attains FEF during the follow up due to their decreased survival rate to reach at full enteral feeding and an earlier death. LBW neonates who were on respiratory support attains FEF at 7 (95% CI: 6–8) and those who didn’t need respiratory support attains at their median age of 3 (3–3) days (Fig. 4).

**Fig. 5:**
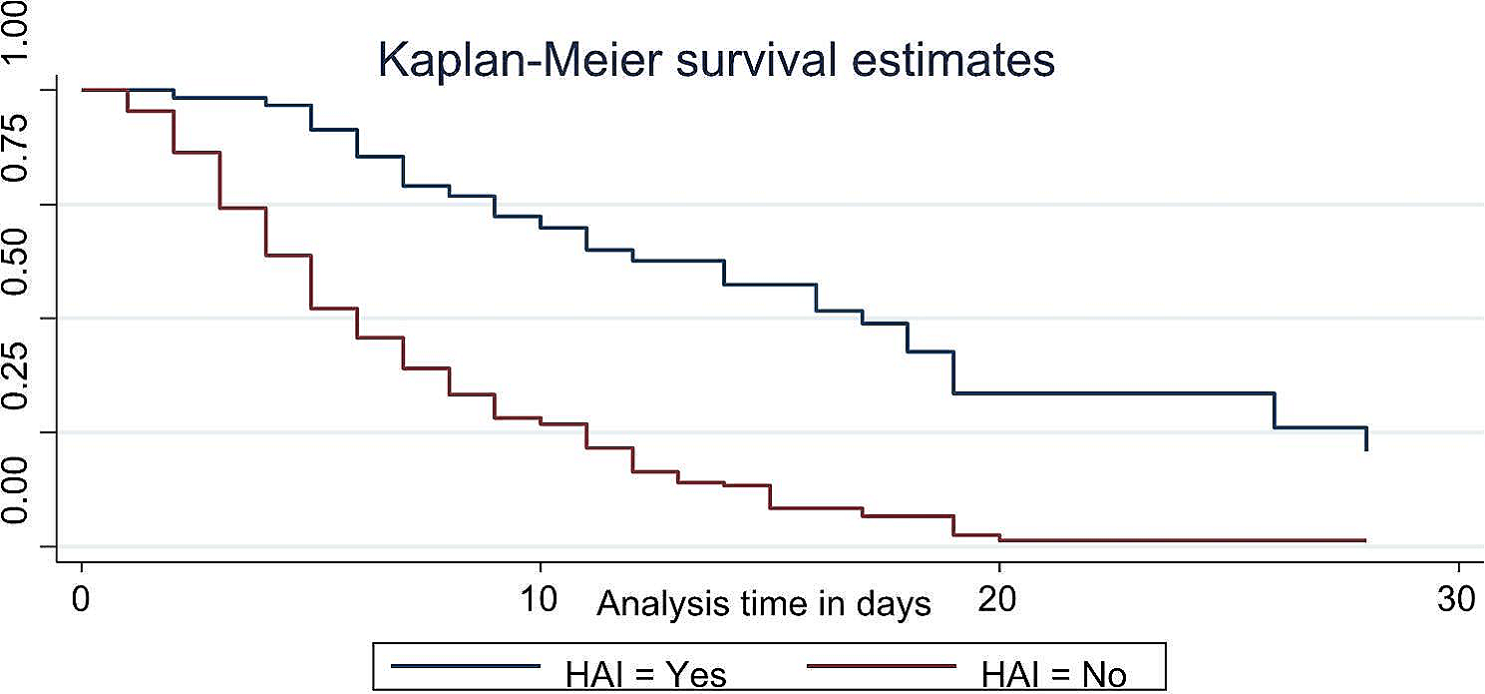
The Kaplan-Meier estimate of time to event (full enteral feeding) curve used to estimate the time to full enteral feeding of low birth weight neonates based HAI during the follow up

**Fig. 6:**
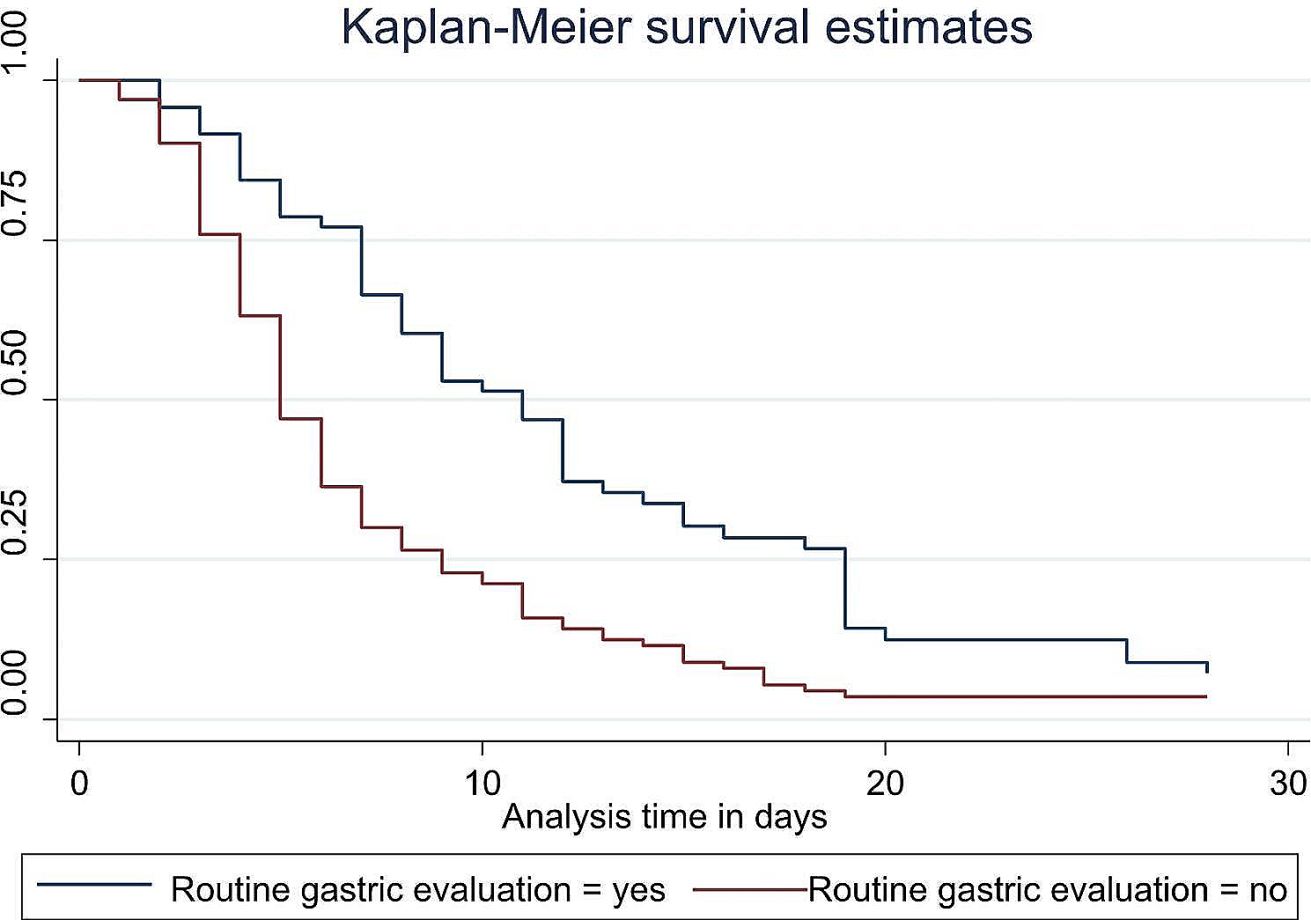
The Kaplan-Meier estimate of time to event (full enteral feeding) curve used to estimate the time to full enteral feeding of low birth weight neonate with pre-feed gastric evaluation

Based on this study’s finding there is also a strong variation in time to FEF based on some clinical condition of the neonates which is newly developed after admission like hospital acquired infection during follow up (Fig. 5) and pre feed gastric evaluation (Fig. 6).

### Predictors of time to time to attain full enteral feeding in LBW neonate admitted to neonatal intensive care units

Maternal Higher educational level (AHR 2.17 and 95% CI 1.012–4.66), birth weight of 1000–1499 gram (AHR 0.31 and 95% CI 0.142–0.67), cesarean delivery (AHR 0.59 and 95% CI 0.36–0.96), hospital acquired infection (AHR 2.25 and 95% CI 1.15–4.41), being on antibiotics (AHR 2.92 and 95% CI 1.31–6.53), age at initiation of trophic feeding, routine gastric residual evaluation (AHR 1.7 and 95% CI 1.1–2.87) and NICU location (SPHMMC) (AHR 4.85 and 95% CI 1.51–15.6) were found to have a statistically significant association with time to attain full enteral feeding in LBW neonates with *p*-Value < 0.05. Maternal educational level was the only maternal socio-demographic characteristics found to have statistically significant association with the time to FEF and this shows neonates whose indexed mothers were at higher educational level 2.17 times more likely to attain full enteral feeding at a given time frame than those whose indexed mothers have no formal education.

Based on the output of multivariate cox regression birth weight of 1000–1499 gram (AHR: 0.31), TF initiation within the first 3 days of postnatal age (AHR: 6, 3.9 and 3.2) respectively were statistically significant predictors with *p* value < 0.05. This indicates that those whose birth weight was in the very low birth weight range (1000–1499 gram) the hazard to FEF at a given time was reduced by 69% when it was compared to their counterparts (95% CI: 0.13–0.62) and early initiation of trophic feeding in the first 24, 24–48 and 48–72 h of birth are six, four and three times more likely to attain full enteral feeding at a given time frame than those who starts TF then after (Table 4).

**Table 4:** Multivariate Cox regression model for predictors of time to attain FEF in LBW neonates admitted to neonatal intensive care unit of Addis Ababa public hospitals, Ethiopia, 202

Covariates	Categories	FEF (%)	Censored (%)	CHR	AHR	*P*-value	95% CI for AHR
Educational level	No education	52 (24.64)	30 (42.25)	**1**	1		
	Primary	56 (26.54)	15 (21.12)	1.27	0.78	0.541	0.405 1.52
	Secondary	44 (20.85)	6 (8.45)	2.2	2.05	0.030*	1.08 4.68
	Technical	19 (9.0)	10 (14.08)	1.5	1.22	0.509	0.436 3.45
	Higher	40 (18.9)	10 (14.08)	2.22	2.17	0.047*	1.012 4.6
Gestational age	28–31	40 (19)	26 (36.6)	0.29	0.82	0.718	0.276 2.4
	32–37	134 (63.5)	32 (45)	0.636	0.91	0.835	0.38 2.16
	≥ 37	35 (16.5)	8 (11)	1	1		
Birth weight in gram	2000–2499	75 (35.5)	17 (24)	1	1		
	1500–1999	79 (37.4)	21 (29.5)	0.65	0.601	0.150	0.30 1.2
	1000–1499	57 (27)	24 (33.8)	0.358	0.31	0.003*	0.142 0.67
Age at admission	Within 1 h	98 (46.4)	39 (55)	1	1		
	1–6 h	76 (36)	20 (28.2)	1.37	0.68	0.156	0.40 1.15
	6–24 h	9 (4.3)	2 (3)	0.89	2.38	0.090	0.87 6.5
	after 24 h	28 (13.3)	10 (14)	1.05	0.48	0.186	0.16 1.41
Delivery - mode	SVD	104 (49)	42 (59)	1	1		
	Cesarean section	106 (50)	28 (39.4)	1.3	0.59	0.034*	0.36 0.96
Hypothermia	Yes	116 (55)	40 (56)	1	1		
	No	95 (45)	31 (44)	1.18	1.37	0.285	0.789 2.23
Respiratory distress	Yes	146 (69.2)	63 (88.7)	1	1		
	No	65 (30.8)	8 (11.3)	1.92	1.16	0.623	0.637 2.11
New diagnoses During follow up.	Yes	135 (64)	63 (88.7)	1	1		
	No	76 (36)	8 (11.3)	3.91	1.78	0.063	0.933 3.15
HAI during follow up	Yes	31 (15)	30 (42)	1	1		
	No	180 (85)	41 (58)	3.52	2.25	0.017*	1.15 4.41
Antibiotics	Yes	177 (84)	70 (98.6)	1	1		
	No	34 (16)	1 (1.4)	4.35	2.92	0.009*	1.31 6.53
Respiratory support	Yes	156 (74)	68 (95.7)	1	1		
	No	55 (26)	3 (4.3)	4.08	4.49	0.236	0.37 54.13
TF initiation age	Within 24 h	53 (23.1)	3 (4.22)	7.12	6.05	0.000*	2.48 14.7
	24-48 h	57 (27)	6 (8.45)	3.47	3.94	0.000*	1.84 8.45
	48-72 h	43 (20.4)	6 (8.45	2.15	3.21	0.001*	1.591 6.51
	After 72 h	27 (13)	9 (12.7)	1	1		
Routine Pre-feed gastric residual evaluation	Yes	59 (32.7)	12 (50)	1	1		
	No	121 (67.3)	12 (50)	2	1.70	0.046*	1.1 2.87
Pacifier	Yes	12 (5.6)	1 (1.4)	1	1		
	No	199 (94.3)	70 (98.6)	0.582	0.564	0.376	0.159 2.00
Hospital	TASH	31 (14.7)	25 (35.2)	1	1		
	ZMH	30 (14.22)	11 (15.5)	1.14	2.43	0.142	0.742 7.98
	GMH	50 (23.7)	7 (9.8)	1.32	2.55	0.083	0.886 7.3
	M II RH	25 (11.8)	13 (18.3)	0.96	1.46	0.589	0.365 5.88
	St. Peter hospital	32 (15.16)	5 (7.04)	1.4	1.7	0.351	0.557 5.18
	SPHMMC	43 (20.38)	10 (14.1)	2.34	4.85	**0.008**	1.51 15.6

AHR, Adjusted hazard ratio; CHR, Crud hazard ratio; FEF, Full enteral feeding; SVD, Spontaneous vaginal delivery; TASH, Tikur Anbessa specialized Teaching hospital; ZMH, Zewditu memorial hospital; GMH, Ghandi memorial hospital; M II RH, Menelik II Referral hospital; SPHMMC, St. Paul’s Hospital Millennium Medical College

## Discussion

This prospective follow up study was aimed to estimate time to full enteral feeding attainment and its predictors among low birth weight neonates admitted to selected public hospitals of Addis Ababa, Ethiopia. Although it has strong variation depending on GA and birth weight the median time to attain full enteral feeding in this study was 5 days. This indicates that they achieve full enteral feeding with in a shorter time when they compared with Hawassa city, Sidama region Ethiopian [30] with a comparable study subjects.

According to this study, 70.1%, 51.8%, 33.4% and 28% of neonates were kept NPO in their 1st, 2nd, 3rd and 4th postnatal day respectively getting only 10% dextrose intravenously. This finding in the first day is almost similar with the results of a hospital-based multicenter prospective study in Ethiopia (76%) [11] but high in the subsequent days. This variation may be due to a difference in the number of study participant involved and study design.

The highest hazard time was the first seven days of life in which majority (64.93%) of LBW were reached at FEF. More than 22% of LBW neonates died before reaching full feeds. Maternal educational level, birth weight, mode of delivery, hospital acquired infection, being on antibiotics, age at initiation of trophic feeding, routine gastric residual evaluation and NICU location were found to have a statistically significant association with time to attain full enteral feeding.

Neonates with birth weight of 2000–2499 gram attains FEF at median postnatal day of 3 while neonates with birth weight of 1500–1999 and 1000–1499 gram attains FEF at 6 and 11 days respectively (Table 3). This result strongly varies with a study done in 13 NICU worldwide (with only one NICU in Africa) which shows the median time to FEF (TFF; 8–33 days) [31]. This variation might be due to availability of TPN in these 13 NICU worldwide (administered for a longer time, 13–25 days) and there is no need to rush for enteral feeding. The other possible explanation for this variation is a difference in the mean GA and birth weight of the study subjects (33.6 ± 2.7 weeks and 1700 ± 426 gram in our case).

However, the median time to attain FEF for VLBW neonates (11 days) was relatively similar with the finding of a population-based retrospective cohort study conducted among VLBW (< 1500 gram) in Bologna, Italy in 2014 with an estimated median time to FEF was 12.9 days (IQR = 8.0–21.5 days) [22]. The difference in our case is no infant less than 1000 gram attains FEF in the neonatal period. The time to FEF for VLBW (< 1500 gram) neonates observed in this study is exactly the same with the finding of studies done in India, 11 days [19]. This might be due to similarity in the study designs and socio-demographic similarity in the study participants.

Maternal educational level was an independent socio-demographic factor in this study, and it had a substantial impact on the time to attain FEF. Neonates whose indexed mothers have a higher education level attains FEF faster than those neonates whose indexed mothers have no formal education. This might be due to poor education/counselling regard to feeding and better awareness on early initiation of feeding among mothers with higher educational level as supported by a study in china [32]. Cesarean delivery delays time to FEF at any time during the follow up by 41% (AHR 0.59 and 95% CI 0.36–0.96) which might be because of CS impairs early breastfeeding activity and a wide range of routine care after CS has become a hindrance to breastfeeding. Neonates without hospital acquired infection (AHR 2.25 and 95% CI 1.15–4.41), are 2.25 times more earlier to attain FEF at any time period during the follow up which was also supported by a study in India in 2018 [19].

Birth weight and Gestational age of neonates are also a significant predictors of time to reach FEF. Neonates with a birth weight of 100–1499 gram (AHR O.31 and 95% CI 0.142–0.67) were up to 69% less likely to attain FEF at any time during the follow up than those neonates whose birth weight is between 2000 and 2499 gram and this is consistent with studies in India [19]. Each 1 week increment in GA leads to 17% increase in the hazard of FEF which is nearly similar with the study done in Bologna, Italy [22].

The timing of initiation of trophic feeding was a significant predictor of time to reach FEF and as the timing of initiation delays, time to FEF also significantly prolonged. Those who start TF in their immediate postnatal day and 2nd were 6 and 3.9 times more faster to reached at FEF than those who starts TF at their 4th day and then after. This is supported by the evidence that starting TF, improves feeding tolerance, significantly fewer days of parenteral nutrition, and oxygen supplementation, and consistently earlier discharge [33].

Despite lack of clear evidence, the practice of routine pre-feed gastric residue aspiration before the next feed is common. Some units and healthcare providers routinely measures the gastric residue volume of every neonate feeding by tube irrespective of the presence of symptom of feeding intolerance. Routine gastric residual evaluation was done for 25.2% of LBW neonate before their next feeding and those who were not on routine gastric residual evaluation were 1.7 times more likely to reached at FEF earlier (AHR 1.7 and 95% CI 1.1–2.87). This is supported by a systematic review and meta-analysis which concludes as avoiding routine pre-feed aspiration was associated with achieving full enteral feeds earlier and shorter duration of hospitalization [34].

Those who were not on antibiotics (AHR 2.92 and 95% CI 1.31–6.53) reaches FEF faster which might be due to the reason that sepsis predicts time to FEF. NICU location (SPHMMC, AHR 4.85 and 95% CI 1.51–15.6) were found to have a statistically significant association with time to attain full enteral feeding which might be due to a difference in feeding practice among hospitals and health professionals and lack of consistent and standard feeding protocol.

### Strengths and limitations of the study

The study was conducted prospectively which can increase quality of data. Data were not missed due to chart incompleteness and it has no conflicts of interest. Study participants were recruited from different health care institution and as a result, the findings can be generalized. As a limitation the study period was short and the health care institution involved in this study were in a similar geographic region where most of the residents are urban.

### Conclusion and recommendation

This study demonstrated the difficulty of understanding which low birth weight neonate attain FEF in a timely manner and identifies some predictors of the time to FEF achievement in LBW neonates. For countries like Ethiopia where 10% glucose is the only available parenteral supplementation and total parenteral nutrition is difficult to afford, this median time to attain FEF is thought to be considered as delayed. Maternal Higher educational level, birth weight of 1000–1499 gram, CS delivery, hospital acquired infection, being on antibiotics, age at initiation of trophic feeding, routine gastric residual evaluation, and NICU location, were found to have a statistically significant association with time to attain full enteral feeding in LBW neonates.

Health care staffs treating LBW neonates should consider preterm nutrition as part of the management and early initiation of trophic feeding should be practiced and concern should be given for prevention of HAI which delays time to FEF and further research is better to be conducted; by taking long follow up time and in each GA and BW category.

## Data Availability

The data extraction tool and data sets used and/or analyzed during the current study are available from the corresponding author on reasonable request and it is also submitted to Addis Ababa University for repository.

## Abbreviations

AGA: Appropriate For Gestational Age
AKI: Acute Kidney Injury
BW: Birth Weight
CPAP: Continuous Positive Airway Pressure
CS: Cesarean Section
EONS: Early Onset Neonatal Sepsis
FEF: Full Enteral Feeding
GA: Gestational Age
HAI: Hospital Acquired Infection
INO2: Intra Nasal Oxygen
EUGR: Extra Uterine Growth Restriction
IYCF: Infant and Young Child Feeding
LBW: Low Birth Weight
LOS: Late Onset Sepsis
NEC: Necrotizing Enterocolitis
NICU: Neonatal Intensive Care Unit
NPO: Nothing Per Mouth
PIH: Pregnancy Induced Hypertension
PNA: Perinatal Asphyxia
RD: Respiratory Distress
SVD: Spontaneous Vaginal Delivery
TEF: Trachea Esophageal Fistula
TF: Trophic Feeding
TFF: Time to Full Feeding
TPN: Total Parenteral Nutrition
VLBW: Very Low Birth Weight
